# Decrease of Intracellular Glutamine by STF-62247 Results in the Accumulation of Lipid Droplets in von Hippel-Lindau Deficient Cells

**DOI:** 10.3389/fonc.2022.841054

**Published:** 2022-02-09

**Authors:** Mathieu Johnson, Sarah Nowlan, Gülsüm Sahin, David A. Barnett, Andrew P. Joy, Mohamed Touaibia, Miroslava Cuperlovic-Culf, Daina Zofija Avizonis, Sandra Turcotte

**Affiliations:** ^1^Department of Chemistry and Biochemistry, Université de Moncton, Moncton, NB, Canada; ^2^Atlantic Cancer Research Institute, Moncton, NB, Canada; ^3^National Research Council of Canada, Digital Technologies Research Center, Ottawa, ON, Canada; ^4^Goodman Cancer Research Centre, McGill University, Montréal, QC, Canada

**Keywords:** metabolomics, glutamine (Gln), CCRCC kidney cancer, von Hippel – Lindau, fatty acid, cancer, lipid droplet (LD)

## Abstract

Kidney cancer is one of the top ten cancer diagnosed worldwide and its incidence has increased the last 20 years. Clear Cell Renal Cell Carcinoma (ccRCC) are characterized by mutations that inactivate the von Hippel-Lindau (VHL) tumor suppressor gene and evidence indicated alterations in metabolic pathways, particularly in glutamine metabolism. We previously identified a small molecule, STF-62247, which target VHL-deficient renal tumors by affecting late-stages of autophagy and lysosomal signaling. In this study, we investigated ccRCC metabolism in VHL-deficient and proficient cells exposed to the small molecule. Metabolomics profiling using 1H NMR demonstrated that STF-62247 increases levels of glucose, pyruvate, glycerol 3-phosphate while glutamate, asparagine, and glutathione significantly decreased. Diminution of glutamate and glutamine was further investigated using mass spectrometry, western blot analyses, enzymatic activities, and viability assays. We found that expression of SLC1A5 increases in VHL-deficient cells treated with STF-62247, possibly to stimulate glutamine uptake intracellularly to counteract the diminution of this amino acid. However, exogenous addition of glutamine was not able to rescue cell viability induced by the small molecule. Instead, our results showed that VHL-deficient cells utilize glutamine to produce fatty acid in response to STF-62247. Surprisingly, this occurs through oxidative phosphorylation in STF-treated cells while control cells use reductive carboxylation to sustain lipogenesis. We also demonstrated that STF-62247 stimulated expression of stearoyl-CoA desaturase (SCD1) and peripilin2 (PLIN2) to generate accumulation of lipid droplets in VHL-deficient cells. Moreover, the carnitine palmitoyltransferase 1A (CPT1A), which control the entry of fatty acid into mitochondria for β-oxidation, also increased in response to STF-62247. CPT1A overexpression in ccRCC is known to limit tumor growth. Together, our results demonstrated that STF-62247 modulates cellular metabolism of glutamine, an amino acid involved in the autophagy-lysosome process, to support lipogenesis, which could be implicated in the signaling driving to cell death.

## Introduction

Kidney cancer affects about 431,000 people worldwide and represents 3% of all malignancies (1). Clear cell Renal Cell Carcinoma (ccRCC) originates from renal tubular epithelial cells and accounts for 75% of RCC diagnosis (2). The remaining 25% consist of papillary RCC (15%), chromophobe RCC (5%), and oncocytomas (5%) (3). Unfortunately, these tumors are asymptomatic leaving one-third of patients with metastases at diagnosis. Moreover, 30-40% of patients with localized tumors relapse following surgery (4). Despite the availability of targeted therapies and the approval of immune checkpoint inhibitors, metastatic RCC 5-year survival rate remains low due to intrinsic or acquired resistance (4, 5). ccRCC are characterized by an early loss of chromosome 3p followed by inactivating mutations affecting the von Hippel-Lindau (VHL) tumor suppressor gene that occurs in up to 85% of cases (6–9). The tumor suppressor function of VHL has been demonstrated in nude mice where restoration of its function inhibits tumor formation (10). In addition, VHL loss served as a building block for additional branch mutations observed on the chromatin-remodeling genes PBRM1 (30-40%), SETD2 (8-12%), BAP1(6-8%) (11–14). In the context of VHL loss, or under hypoxic conditions, the hypoxia-inducible factor alpha (HIFα) is stabilized which activates the transcription of genes that regulate oncogenic transformation, angiogenesis, and metabolism (15, 16). The VHL-HIF axis is the foundation of targeted therapies for metastatic RCCs with drugs (e.g., sunitinib, pazopanib, axitinib) that target VEGFR (17).

Metabolic reprogramming has been recognized as a hallmark of cancer (18). Hypoxic conditions observed in tumor microenvironment increased glycolysis supporting cell proliferation (19–21). In addition to HIF-1α and HIF-2α activation, cancer-causing mutations can also directly alter metabolism to promote glutamine utilization. For example, the oncogene c-Myc activates glutaminolysis by repressing miR23a/b while K-Ras mutations stimulate aspartate formation derived from glutamine (22, 23). For a long time, ccRCCs have been known as a metabolic disease. In fact, it has been shown that VHL mutations induce a metabolic switch promoting glutamine reductive carboxylation instead of supporting oxidative phosphorylation to support lipogenesis (24, 25). Glutamine is a non-essential amino acid (NEAA) synthesized *de novo* and metabolized by all cells to support the biosynthesis of nucleotides, glutathione and other NEAA. Glutamine and derivatives have been implicated in epigenetic regulation, such as histone post-translational modifications and DNA methylation levels and can also contribute to resistance to targeted therapy (26, 27). Glutamine is incorporated into the cells through the Solute Carrier (SLC) membrane transporter SLC1A5 as well as others from the SLC6, SLC7 and SLC38 transporter families (28). High expression of SLC1A5 has been reported in several cancers including ccRCC (29–31). Inside mitochondria, glutamine is converted into glutamate by the glutaminase (GLS1), which can then enter Krebs cycle. Inhibitors of GLS1 have been shown to repress cancer growth in tumors addicted to glutamine (24, 25, 32, 33). Finally, glutamine goes through reductive carboxylation to form citrate and support fatty acid production, particularly in VHL-deficient RCC when HIF-2α is overexpressed (24). Changes in fatty acid (FA) metabolism have been documented in ccRCC and accumulation of lipid droplets in the cytosol is a phenotypic characteristic of these tumors (34). For example, it has been shown that HIF-α represses the expression of carnitine palmitoyltransferase 1A (CPT1A) responsible for β-oxidation in the mitochondria (35). In addition, peripilin 2 (PLIN2) and stearoyl-CoA desaturase (SCD1) are highly expressed in HIF-2α dependent ccRCC, both favoring lipid storage (36, 37).

We previously performed metabolomic profiling to identify intracellular and extracellular deregulated metabolites in VHL-inactivated cells compared to cells with the functional gene and we used shRNA against HIF-1α and HIF-2α to further describe those regulated by HIF-α (38). Here, we evaluated the effect of STF-62247 on ccRCC metabolism. STF-62247 is a small molecule, cytotoxic to VHL-inactivated ccRCC compared to cells stably expressing VHL, that we previously identified from a screen (39). We showed that this small molecule affects the autophagic flux by targeting lysosome vulnerabilities observed in tissue from patients with ccRCC (40, 41). Here, we showed that STF-62247 decreased significantly intracellular levels of glutamine and glutamate in VHL-deficient cells without any change in GLS1 activity. However, addition of extracellular glutamine into the media is not sufficient to rescue STF-62247 treated cells. Surprisingly, metabolic flux analysis of glutamine indicated that VHL-deficient cells exposed to the small molecule favor the oxidative tricarboxylic acid (TCA) cycle to produce citrate and increase FA production in contrary to control cells that use reductive carboxylation to support lipogenesis. Finally, we observed that SCD1 and PLIN2 expression, which stimulate the formation of lipid droplets into VHL-deficient RCC, increased in response to STF-62247.

## Materials and Methods

### Cell Culture

The human ccRCC cell lines (RCC4, RCC10 and 786.0) and their isogenic counterparts stably expressing VHL (RCC4/VHL, RCC10/VHL and 786.0/VHL) were kindly provided by Amato J. Giaccia (Stanford University, CA, USA). All cell lines were tested for mycoplasma and authentication was performed by short tandem repeat (STR) DNA profile at Genetica DNA Laboratories (Burlington, NC, USA). Cells were maintained in DMEM/high glucose medium (GE Healthcare Life Science, UT, USA), supplemented with 10% Fetal bovine serum (FBS) (Wisent Bio Science, QC, Canada), 2 mM L-glutamine and 1mM sodium pyruvate (GE Health Life Sciences, ON, Canada) and cultured at 37˚C in a humidified incubator with 5% CO_2_.

### Cell Treatment and Metabolite Extraction

Cells were treated with STF-62247 (Cayman Chemical) at 1.25 μM STF-62247 for 24 hr in RCC4 and RCC4/VHL cells and 2.5 μM for 48 hr in 786.0, 786.0/VHL, RCC10 and RCC10/VHL cells. Cells were harvested with the media by scraping and centrifuged at 1,500 x g for 1 min at 4°C. The pellet was washed with cold PBS and then resuspended in 1 mL ice-cold acetonitrile:water solution (1:1). Samples were incubated on ice for 10 min and centrifuged at 16,000 x g for 10 min at 4°C and the supernatants were placed at -80°C.

### NMR Experimentation, Data Analysis and Metabolite Quantification

The extract supernatants were dried under a stream of N_2_ and dissolved in 0.7 mL deuterium oxide (Aldrich, 99.96% atome ^2^H). All ^1^H NMR analysis were performed on a Bruker Avance III 400 MHz at 298K and the spectra were obtained using a gradient water presaturation method with 512 scans as previously described (38). Spectra were processed using the Mnova software with exponential apodization, global phase correction, Berstein-polynomial baseline correction and Savitzky-Golay line smoothing and normalization using total spectra area and regions from 0.5 to 4.5 ppm and 5 to 9 ppm were included in the normalization and analysis. Principal component analysis was performed through Matlab vR2010b platform and hierarchical clustering was done with TMeV software with the significance analysis for microarray (SAM) method. Peak assignment was processed through several methods developed in our group and elsewhere based on NMR databases (www.hmdb.ca and www.bmrb.wisc.edu). An automated method for quantification based on multivariable linear regression of spectra with aligned standard metabolite data from databases was developed and used previously.

### Glutamine, Glutamate and Leucine Quantification Using LC-MS Analysis

The extract supernatants were filtered through 300 kDa molecular weight cut-off filters (Pall, Ann Arbour, Michigan) at 14,000 x g for 20 min at 4°C to remove any particulates. The flow-through from all samples was then diluted 20-50 fold in acetonitrile:water (9:1) before injection on the LC-MS platform. All samples were spiked with 1 μM of internal standard. The liquid chromatograph was an Agilent 1100 (Agilent, Palo Alto, CA). A Sequant^®^ ZIC-cHILIC chromatographic column (EMD MilliporeCorp. Billerica, MA) was used (150 mm long and 2.1 mm in diameter). The column was packed with 3μm diameter particles with a pore size of 100 Å. The addition of 20 mM ammonium acetate to solvent B (water) was found to be critical for analyte retention and separation. The column flow rate was 100 μL/min, the column back pressure was approximately 180 bar and the sample injection volume was 5 μL. Solvent A was acetonitrile and solvent B was 20 mM aqueous ammonium acetate. The solvent gradient was ramped from 10 - 30% B in 1 min, then to 41% B over 15 min and finally back to 10% B for 24 min to re-equilibrate the column. The observed retention times were as follows: Gln/d5-Gln (22.68 minutes), Glu/d5-Glu (22.25 minutes), Leu/d10-Leu (16.85 minutes) and Ile (17.41 minutes). Note that leucine and isoleucine are nearly baseline resolved. Chromatographic peak widths were roughly 12-15 sec. HR/AM mass spectrometric detection was accomplished with a hybrid quadrupole/Orbitrap mass analyzer (Q-Exactive, Thermo-Fisher Scientific, San Jose, CA) operated in negative ion mode at a resolution of 140,000. The mass-to-charge scan range was 100-500. Measured molecular weights for endogenous glutamine, glutamate and leucine as well as their deuterated analogs were all within 5 ppm of their calculated weights. The observed mass-to-charge values were as follows: Gln (145.0603), Glu (146.0444), d5-Gln (150.0919), d5-Glu (151.0759), Leu (130.0858) and d10-Leu (140.1486). Calibration curves for the LC-MS system were established for a concentration range of 1 nM to 25 μM using the isotopically labeled compounds as internal standards at a level of 1 μM throughout. Interface conditions on the Q-Exactive were as follows: Sheath, auxiliary and sweep gas flow rates (7, 1 and 1 arbitrary units); ionspray voltage (3.5 kV), heated capillary voltage (320°C) and S-lens voltage (50 V). Data was processed using the Quan Browser node of the Xcalibur 3.0.63 software. Calibration curves of analyte peak area relative to that of the appropriate internal standard were plotted as a function of analyte concentration.

### Western Blot Analysis

Total proteins were extracted using M-PER lysis buffer (50mM Tris (HCl) pH 7.5, 200 mM NaCl, 0.25% Triton X-100, 10% Glycerol) containing 1X protease and phosphatase inhibitor cocktails. Cell lysates were centrifuged at 12,000 x g for 10 min at 4°C and quantified by BCA protein assay kit (Thermo Fisher scientific) using the SpectraMax Plus 384 microplate reader. Proteins (30µg) were separated on 10-15% SDS-PAGE gel and transferred onto a 0.45-micron PVDF transfer membrane (Immobilon-P, Millipore, IE). Membranes were blocked using 5% skim milk diluted in a solution of 0.075% PBS-Tween (PBS-T) and incubated overnight in 3% BSA with specific primary antibodies against VHL, HIF-1α and HIF-2α (Cell signaling #68547, 14179, 59973), SLC1A5 and β-actin (Santa Cruz Biotechnologies sc-99002 and sc-47778), SLC7A5 (Medical & Biological Laboratories #BMP011), SLC3A2 (Aviva Systems Biology #OAAB00158), ASNS (Signalway #32909), SCD1 (Applied Biological Materials #ABM-G076), PLIN2 (Proteintech #15294-1-AP) and CPT1A (Abcam #ab220789). Immunoblots were washed and incubated with HRP-conjugated secondary antibodies diluted in 5% skim milk PBS-T (Jackson Immunoresearch Laboratories, PA, USA) and visualized using the ClarityTM ECL substrate, (Bio-Rad, ON, Canada) on a Chemidoc MP Imager (Bio-Rad).

### Glutaminase and Glutamine Synthetase Activity

For glutaminase activity, 10 μg of protein was mixed with 10 μL of buffer A (50 mM Tris-acetate pH 8.6, 20 mM glutamine, 100 mM K_2_HPO_4_, 0.2 mM EDTA) and incubated at 37°C for 10 min. Reaction was stopped with 2 μL of HCL 2M. Then, 200 μL of Buffer B (80 mM Tris-acetate pH 9.4, 200 mM hydrazine, 0.25 mM ADP, 2mM NAD and 2.5 U Glutamate deshydrogenase) was added to the sample and absorbance was read at 340 nm on a SpectraMax i3 (Molecular Devices) each 1 min for 5 min and then at 5 min interval for 30 min. GLS activity was calculated according to the Beer’s Law (dAbs/ε*d)(10^6^)(V_t_/V_s_) where ε = molar extinction coefficient of NADH, d = path length of light through the sample, Vt/Vs= total volume/sample volume. To measure activity of the glutamine synthetase, 40 μg of proteins in 50 μL of lysis buffer (50 mM imidazole-HCl pH6.8) were mixed with 50 μL of reaction buffer (50 mM imidazole pH 6.8, 50 mM glutamine, 25 mM hydroxylamine, 25 mM sodium arsenite, 2 mM MnCl_2_, 0.16 mM ADP) and incubated for 4 hr at 37°C. Then, 100 μL of stop solution (2.42% FeCl_3_, 1.45% TCA, 1.82 N HCl) was added and products were centrifuged at 16,000 x g at 4°C for 10 min. Supernatants were transferred into a microplate and absorbance was read at 560 nm. A standard curve of γ-glutamylhydroxamate was generated and used to calculate GS activity and reported in nmol/min/g protein.

### Mitochondrial Oxygen Consumption Experiments

Cell respiration was performed on a Oxygraph-2K (Oroboros instrument) in a glass chamber. Cells were trypsinized and 5 x 10^5^ cells/mL were added to the chamber and the respiration was measured at the basal level to represent the endogenous respiration state of the cells. The non-coupled resting respiration was measured after adding 2.5 μM of oligomycin followed by the gradual addition of FCCP (up to 2 μM) to evaluate the maximal uncoupled capacity of the electron transport system (ETS). Finally, 2.5 μM antimycin A and 0.5 μM rotenone were added to measure the residual oxygen that occurs independently of oxidative phosphorylation. Respiratory control ratio (RCR) was calculated from the ratio between the uncoupled and non-coupled respiration.

### Cell Proliferation, Clonogenic and XTT Assays

Growth curves were performed in 12 well plates with 30,000 cells in duplicate. STF-62247 (1.25 μM), glutamine (10 mM), asparagine (2 mM) or lysophosphatidylcholine 18:1 (25 μM) (Avanti Polar Lipids, Inc.) was added 12 hr after plating. Cells were trypsinized and counted with trypan blue at different time points. For clonogenic assays, 300 cells were seeded in triplicate into 60 mm plates overnight and STF-62247 (0-5 μM) was added to the cells. Plates were incubated at 37°C for 8 days. Colonies were fixed and stained with a solution of crystal violet and quantified. For XTT assays, 5,000 cells were seeded in duplicate in 96 well plates. Cells were treated with STF-62247, BCH (Tocris Bioscience) and L-Glutamic acid γ-(p-nitroanilide) hydrochloride (Sigma-Aldrich) 12 hr after seeding and plates were incubated for 4 days at 37°C. Then, XTT solution (0.3 mg/mL of XTT powder (Sigma-Aldrich), DMEM high glucose without phenol red (Wisent Bio), 20% FBS and 2.65 μg/mL phenazine methosulfate (PMS) (Sigma-Aldrich)) was added to the cells and incubated at 37°C for 1 hr. Absorbance was read at 450 nm on a Spectramax Plus spectrophotometer (Molecular Devices, Sunnyvale, CA). All these assays were performed in biological triplicate.

### RNA Isolation and Quantitative Real Time-Polymerase Chain Reaction

Total RNA was isolated from cell lines using TRIzol reagent (Thermo Fisher Scientific) according to the manufacturer’s protocol. The RNA was quantified using the Nanodrop ND-1000 (Thermo Fisher Scientific) and its purity was assessed by the ratio absorbance 260/280. RNAs (5µg) were subjected to reverse transcription using SuperScript III reverse transcriptase (Thermo Fisher Scientific). The resulting cDNA was used for real-time qPCR (Realplex^2^, Eppendorf) for quantification by SYBR green (Quanta Biosciences). cDNA was first denaturated at 95°C for 3 min followed by hybridization and elongation at 60°C and 72°C, respectively for 40 cycles. RNA expression was normalized to RPLPO expression. All primers sequences were provided by primerbank (https://pga.mgh.harvard.edu/primerbank/) as follow: SLC1A5 forward 5’-TCATGTGGTACGCCCCTGT-3’ and reverse 5’-GCGGGCAAAGAGTAAACCCA-3’; SLC3A2 forward 5’-TGAATGAGTTAGAGCCCGAGA-3’ and reverse 5’-GTCTTCCGCCACCTTGATCTT-3’; SLC7A5 forward 5’-GTGGACTTCGGGAACTATCACC-3’ and reverse 5’-GAACAGGGACCCATTGACGG-3; RPLPO forward 5’-GCAATGTTGCCAGTGTGT-3’ and reverse 5’-GCCTTGACCTTTTCAGCAA-3’. mRNA relative expression was calculated with the ΔΔCt method.

### Metabolic Flux Studies, ^13^C_5_-Glutamine Labeling and GC-MS Analysis

Cells were plated in DMEM supplemented with 10% dialyzed FBS (Wisent Bio), 1 mM glutamine and 1 mM sodium pyruvate and 1.25 μM STF-62247 was added for 24 hr. For glutamine metabolic tracing, media was replaced with DMEM without glutamine supplemented with ^13^C_5_-L-glutamine (Cambridge Isotopes Laboratories, Tewksbury, MA), 10% dialyzed FBS with or without STF-62247 for up to 2 hr. In addition, dishes were kept in unlabeled media as control. Metabolite extraction was performed at different time points as previously described and samples were sent to McGill Metabolomics Core Facility for GC-MS analysis. Briefly, supernatants were dried by spin vacuum, dissolved in a solution of 10 mg/mL methoxyamine: HCl in pyridine, and derivatized with MTBSTFA for 1 hr at 70°C. One microliter of derivatized samples was analyzed through an Agilent 5975C Series GC/MSD with the Triple-Axis HED/EM Detector coupled to a 7890A gas chromatograph equipped with a 7693 autosampler (Agilent, Santa Clara, CA). Data are collected and analyzed by Chemstation software or Mass Hunter. Spectra are identified using Fiehn, Bains (Steadman Metabolism laboratory, Duke University) or NIST17 databases and authentic samples.

### ^14^C-glutamine labeling and Lipid Synthesis

50,000 cells were seeded in 12-well plates in duplicate, and treated with 1.25 μM STF-62247 for 24 hr. The last hour, media was replaced with DMEM supplemented with 0.2 μCi/mL (L-[^14^C(U)]-Glutamine (American Radiolabeled Chemicals, St-Louis, MO). Lipids were extracted according to a modified version of Bligh and Dyer method (42). Briefly, after trypsinization, a solution of cells (400 µL trypsin and 400 µL PBS for a total of 800 µL) was added to 3 mL methanol and chloroform solution (2:1) followed by the addition of 25 µL of 10% acetic acid. Samples were vortexed and incubated at room temperature for 15 min. Another 2 mL chloroform and 1 mL water were added, in order, before carefully mixing. Samples were centrifuged at 1,000 RPM for 5 min at room temperature. The chloroform phase (on top) was transferred to a new tube and put on the side. Another 2 mL chloroform was added to the samples, mixed and centrifuged like precedently. The chloroform phase was pooled with the first one before being air-dried with nitrogen and resuspended in scintillation liquid. Total extractable counts were obtained using LS 6500 multipurpose scintillation counter (Beckman Coulter™) and normalized with cell number.

### Lipid Droplets Quantification

Cells were seeded in 8 well chamber slides and treated for 48 hr with 1.25 µM STF-62247. On the last day, cells were fixed with 3.7% formaldehyde, lipid droplets were stained with HSC LipidTOX™ green neutral lipid stain (ThermoFisher) according to the manufacturer protocol and nuclei were stained with DAPI. Cells were incubated in LipidTOX (1:3000 dilution) for at least 30 min before images were taken using a 60X objective lens on an Olympus Fluoview FV1000 confocal microscope (Olympus). Images were analyzed, and puncta were counted using Fiji (ImageJ) using three pictures for each experimental condition in three biological replicates.

### Statistical Analysis

Statistical analyses were performed using RStudio (RStudio Team (2020). RStudio: Integrated Development Environment for R. RStudio, PBC, Boston, MA URL http://www.rstudio.com/) and GraphPad Prism version 6.01 for Windows, GraphPad Software (La Jolla, CA) www.graphpad.com. Data for glutamine and glutamate quantification by LC-MS, enzymatic activities, RCR, radiolabeled glutamine-derived fatty acid synthesis and lipid droplets counts were analyzed using a two-way ANOVA for the comparisons of the two independent variables: VHL status (RCC4 VHL- and VHL+) and treatment conditions (CTL and STF-62247). For cell proliferation and viability, the variables were the treatments (CTL, STF-62247 and/or Gln, Asn or LPC 18:1) and the time points (day 1, 3 and 5). Shapiro-Wilk’s and Levene’s tests were then used to verify the normality and homogeneity of variance. When two-way ANOVA results showed interaction between the variables, Tukey’s multiple comparison *post-hoc* test was used to determine significant differences between means. Metabolites fractions from metabolic flux analysis were also analyzed with a two-way ANOVA with the two factors being conditions (CTL and STF-62247) and time points (20, 60 and 120 min). Sidak’s multiple comparisons test was used to compare the significant differences between conditions at each time points (results at 120 min are shown in **Figure 5C**). SLC mRNA levels were analyzed with Student t-test to compare each transporter individually to their respective control (either CTL vs. STF, or RCC4 VHL- vs. RCC4 VHL+). Each experiment has been performed at least three times. When it is applicable, results are presented as the mean and the SEM.

## Results

### Effect of STF-62247 on RCC Metabolism

**Figure 1:**
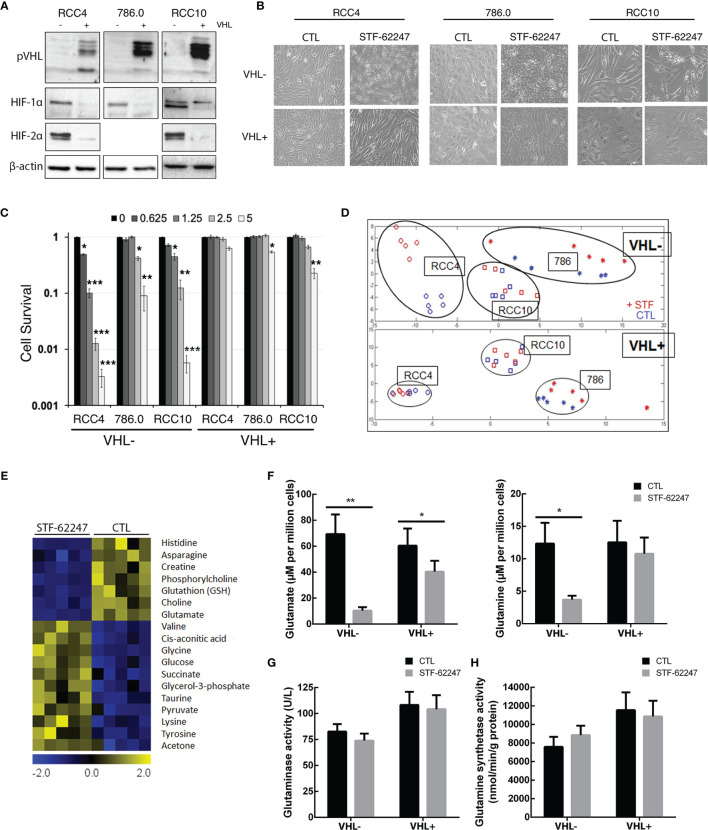
Metabolic profiling in renal cell carcinoma cell lines treated to STF-62247. **(A)** Immunoblot analysis of VHL- RCC cells (RCC4, 786.0 and RCC10) and their counterparts where VHL was reintroduced (indicated by the notation VHL+). Validation of our models was done by detecting pVHL and hypoxia-inducible factors (HIF-1α and HIF-2α). **(B)** Inverted-light microscopy images of vacuolization in VHL- and VHL+ cell lines treated to STF-62247. **(C)** Cell survival was measured by clonogenic assay in VHL- and VHL+ cells. Cells were treated with doses of STF-62247 ranging from 0 to 5 µM. Results are normalized with their respective control (dose 0). Student’s *t*-tests were used to evaluate significant differences for each STF-62247 dose (N=3). **(D)** Principal component analysis (PCA) of ^1^H NMR metabolic data from renal cell carcinoma cell lines. RCC4 cells were treated with STF-62247 at 1.25 μM for 24 hr and 786.0 and RCC10 cells at 2.5 μM for 48 hr (N=5). **(E)** Significance analysis of microarray (SAM) of differentially concentrated intracellular metabolites in RCC4 VHL- cells when treated to 1.25 µM STF-62247 (N=5). **(F)** Intracellular glutamine and glutamate measured by LC-MS in RCC4 VHL- and VHL+ treated with 1.25 µM STF-62247 for 24 hr (N=5). **(G, H)** Enzymatic activity of glutaminase **(G)** and glutamine synthetase **(H)** in RCC4 VHL- and VHL+ cells treated with 1.25 µM STF-62247 for 24 hr (N=3 and N=4 respectively). Results are presented as means and SEM of at least three independent experiments. Two-Way ANOVA followed by Tukey’s test was performed to assess significant differences in the results **(F–H)**. (*p < 0.05, **p < 0.01, ***p < 0.001).

To evaluate the effect of STF-62247 on cell metabolism, we used three different RCC cell lines such as RCC4, 786.0 and RCC10 parental cell lines which harbors different mutations on the VHL gene. The RCC4 cells are characterized by a VHL missense mutation at residue 65 (S65W), while 786.0 cells present a frameshift mutation (G104fs*55) leading to a nonfunctional VHL protein (pVHL) and analysis of VHL sequence in RCC10 cells DNA indicated a deletion mutation of the amino acid 159 lysine introducing a stop codon (43). The wild-type VHL gene has been stably reintroduced into the parental cell lines to generate RCC4/VHL, 786.0/VHL and RCC10/VHL. Indeed, pVHL expression is absent in parental cell lines (VHL-) compared to the VHL reintroduced cells (VHL+) (**Figure 1A**). As expected, VHL mutations in the parental cells lead to stabilization and expression of HIF-2α and HIF-1α, except for 786.0 cells that do not express HIF-1α (**Figure 1A**). A phenotypic characteristic of VHL- cells treated with STF-62247, a small molecule identified to target the loss of VHL, is the presence of intracytoplasmic vacuoles, which we demonstrated to be related to enlargement of endolysosomes (39–41, 44). This phenotype is observed mostly in VHL- RCC4 and RCC10 cells compared to their counterpart VHL+ (**Figure 1B**). In addition, these three cell lines (VHL-) are sensitive to STF-62247 compared to the VHL+ cells, particularly RCC4 cells with a IC50 at 1.25 μM. Although 786.0 cells are less sensitive to STF-62247 and present smaller and lower intracytoplasmic vacuoles, they still show selectivity for the loss of VHL (**Figure 1C**). Thus, a metabolic profiling was generated using ^1^H NMR in the three VHL- cell lines compared to the same cells with the functional VHL gene in response to STF-62247 (**Figure 1D**). Hydrophilic metabolites were extracted from five biological replicates. As previously reported, principal component analysis (PCA) showed altered metabolite patterns in absence or presence of VHL (38). In addition, PCA spectra showed a clear separation in STF-treated cells (STF) compared to control (CTL) in the VHL- RCC4 cell line. A slight alteration in metabolites extracts was observed in STF-treated 786.0 and RCC10 treated cells compared to control. PCA was similar in response to STF-62247 in three VHL+ cell lines (RCC4, 786.0, RCC10). Since RCC4 was the model more sensitive to the small molecule and showing the greatest difference on metabolic profiling, we move forward with this cell line to investigate these changes. Significance microarray analysis (SAM) was used to quantify metabolite significant changes in VHL- cells exposed to STF-62247 (**Figure 1E**). The results demonstrated that glycolysis components such as glucose, glycerol-3-phosphate and pyruvate were significantly increased in treated cells. In the opposite way, glutamate, glutathione (GSH), and asparagine displayed important decreases in response to STF-62247. Because of its implication in ccRCC, the fate of glutamine was the focus of our investigation. To validate ^1^H NMR results, quantification of intracellular glutamine and glutamate levels using LC-MS showed a diminution of these two amino acids of 70% and 85% respectively, in VHL- cells in response to STF-62247 supporting our previous findings (**Figure 1F**). No change in glutamine level was observed in VHL+ cells treated with STF-62247 while glutamate decrease by 33%. VHL inactivation did not significantly influence basal intracellular levels of these two amino acids. Next, we measured the activity of glutaminase, the enzyme responsible for the conversion of glutamine into glutamate, which is highly expressed in the kidney. However, no significant difference was observed on glutaminase activity between VHL- and VHL+ cells or in response to STF-62247 (**Figure 1G**). Finally, we quantified the glutamine synthetase activity, which produces glutamine from glutamate. Here, the results showed higher activity in VHL+ cells compared to VHL- cells and a slight increase in response to STF-62247 (**Figure 1H**). These results demonstrated that STF-62247 decreased glutamine and glutamate levels in VHL- cells without affecting GLS activity suggesting that import of glutamine can be altered, or that cytoplasmic glutamine is highly consumed.

### Are Glutamine Transporters Responsible for the Glutamine Drop?

**Figure 2:**
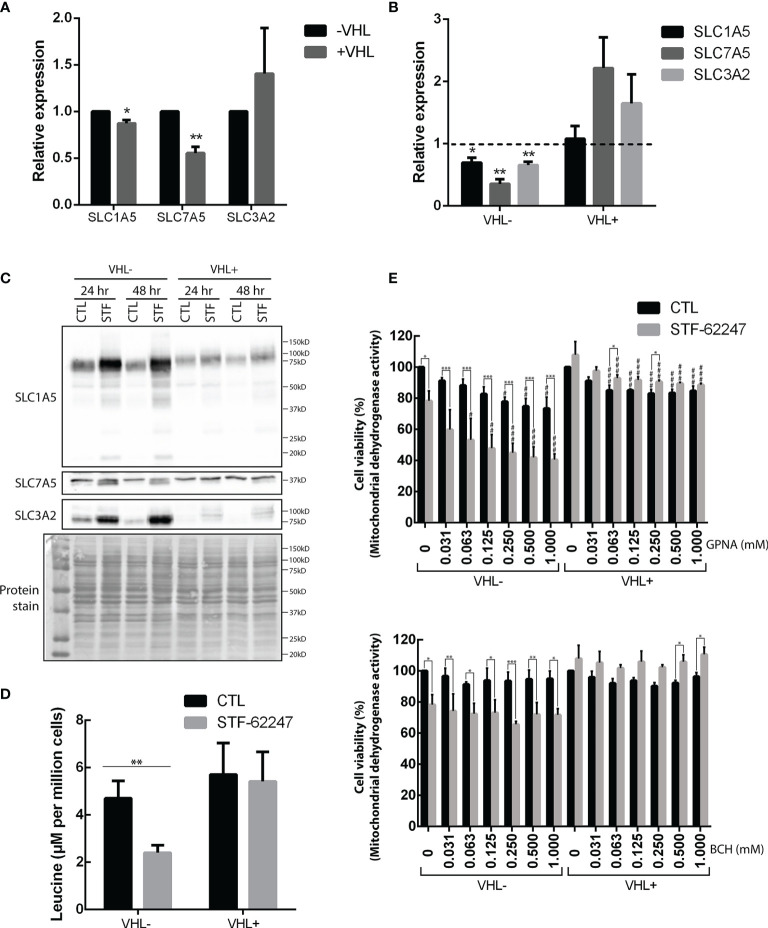
Effects of STF-62247 on glutamine transporters. **(A, B)** mRNA relative expression, measured by RT-qPCR, of glutamine transporters SLC1A5, SLC7A5 and SLC3A2 influenced by **(A)** VHL status and **(B)** STF-62247. RCC4 VHL- and VHL+ were treated with 1.25 µM STF-62447 for 24 hr. Results for treated cells are compared to control cells (represented by the dotted line at 1). Results are presented as means and SEM of three independent experiments. **(C)** Glutamine transporters, SLC1A5, SL7A5 and SLC3A2, protein levels in RCC4 VHL- and VHL+ treated to STF-62247 for 24 and 48 hr. **(D)** Intracellular leucine was measured by LC-MS in RCC4 VHL- and VHL+ treated with 1.25 µM STF-62247 for 24 hr (N=6). Student’s *t*-tests were performed to compare results between VHL- and VHL+ cells **(A)** or between controls and treated cells **(B, D)** (*p < 0.05, **p < 0.01, ***p < 0.001). **(E)** Cell viability was evaluated by XTT assay in RCC4 VHL- and VHL+ cells. SLC1A5 and SLC7A5 inhibitors, GPNA and BCH respectively, were tested alone (concentrations from 0 to 1 mM) or combined with 1.25 µM STF-62247 (N=3). Two-Way ANOVA followed by Tukey’s test was performed to assess statistically significant results. Comparison between CTL and STF-62247 conditions for each concentration of inhibitor (GPNA or BCH) are denoted with the following statistical marks *p < 0.05, **p < 0.01 or ***p < 0.001. Comparison of each inhibitor concentrations (x-axis) are made with their respective control (0 mM columns, with or without STF-62247) and statistical significances are denoted by ^#^p < 0.05, ^##^p < 0.01 or ^###^p < 0.001.

**Figure 3:**
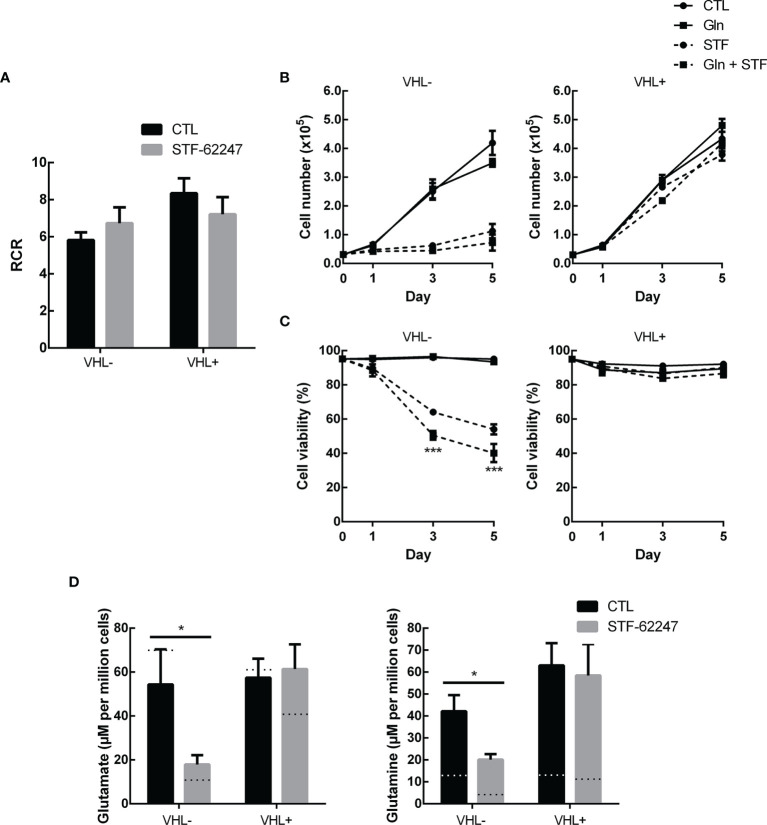
Glutamine excess is not sufficient to overcome stress from STF-62247 **(A)** Respiratory control ratio (RCR) measured with an Oxygraph-2K (Oroboros) in RCC4 VHL- and VHL+ treated with 1.25 µM STF-62247 for 24 hr (N=3). **(B)** Proliferation and **(C)** Viability of RCC4 VHL- and VHL+ treated with 1.25 µM STF-62247 and/or an excess of 10 mM glutamine. Cells were counted on days 0, 1, 3 and 5 with trypan blue to evaluate cells viability (N=3). Statistical marks shown in cell proliferation and viability curves are either between CTL and Gln, or between STF and STF + Gln conditions. **(D)** Intracellular glutamate and glutamine measured by LC-MS in RCC4 VHL- and VHL+. Cells were seeded in media containing 10 mM glutamine and treated with 1.25 µM STF-62247 for 24 hr (N=6). Dotted lines represent values from data obtained from cells incubated in normal media containing 2 mM glutamine (Data from **Figure 1C**). Results are presented as means and SEM of at least three independent experiments. Statistical analyses (Two-Way ANOVA followed by Tukey’s test) were performed to assess significant differences in the results **(A–D)**. (*p < 0.05).

**Figure 4:**
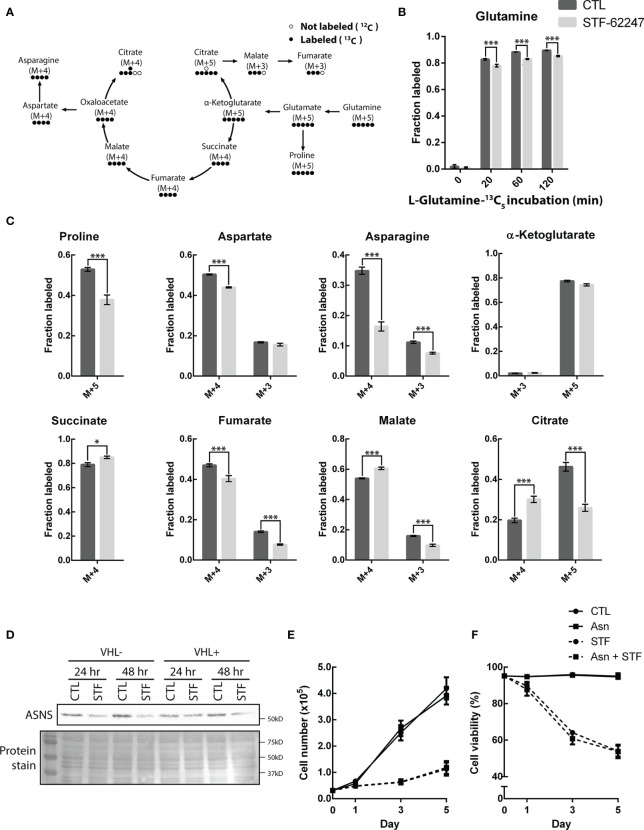
STF-62247 causes a shift in glutamine metabolic flux and increases the use of the oxidative TCA cycle. **(A)** Simplified schematic representation of the carbon exchange in glutamine metabolism. Arrows do not always represent direct metabolic reactions. **(B)** L-Glutamine-13C5 incorporation, assessed by GC-MS, in RCC4 VHL- untreated and treated with 1.25 µM STF-62247 for 24 hr (N=3). **(C)** Metabolites fractions labeled from L-Glutamine-13C5 (120 min incubation) in RCC4 VHL- untreated or treated with STF-62247 for 24 hr (N=3). Metabolites derived from oxidative TCA: Succinate M+4, Fumarate M+4, Malate M+4, Aspartate M+4, Asparagine M+4, Citrate M+4, α-Ketoglutarate M+3. Metabolites derived from reductive TCA: Citrate M+5, Malate M+3, Fumarate M+3, Aspartate M+3 and Asparagine M+3. **(D)** Immunoblot analysis of asparagine synthetase (ASNS) in RCC4 VHL- and VHL+ after 24 hr and 48 hr of STF-62247 treatment. **(E)** Proliferation **(F)** and viability of RCC4 VHL- treated with 1.25 µM STF-62247 and/or 2 mM Asparagine (Asn). Cells were counted on days 0, 1, 3 and 5 with trypan blue to evaluate cells viability (N=3). Statistical marks shown in cell proliferation and viability curves are either between CTL and Asn, or between STF and STF + Asn conditions. Results are presented as means and SEM of three independent experiments. Statistical analyses (Two-Way ANOVA followed by Sidak’s **(B, C)** or Tukey’s test **(E, F)**) were performed to assess significant differences in the results. (*p < 0.05, ***p < 0.001).

Glutamine can be incorporated into the cytoplasm *via* SLC1A5, which can be coupled with SLC7A5 and its chaperone SLC3A2 to exchange glutamine with another amino acid such as leucine (45). First, we quantified mRNA levels of these transporters and showed that SLC1A5 and SLC7A5 are significantly higher in VHL- cells compared to VHL+ and support previous work demonstrating that SLC1A5 and SLC7A5 expression are mediated by HIF-2α (46, 47) (**Figure 2A**). Moreover, our results indicated that mRNA levels of these transporters and the chaperone SLC3A2 significantly decrease in response to STF-62247 in VHL- cells. An opposite effect was seen in VHL+ cells where SLC7A5 and SLC3A2 mRNA levels increased in response to the small molecule although it was not statistically significant, and no effect was observed on SLC1A5 (**Figure 2B**). At the protein level, expression of SLC1A5 and SLC3A2 is also higher in VHL- cells compared to VHL+ cells while SLC7A5 protein expression is similar in both cells. (**Figure 2C**). Curiously, SLC1A5 and SLC3A2 proteins expression was quite contrasting with mRNA levels since both are highly increased, particularly in VHL- cells in response to STF-62247. The presence of several bands for SLC1A5 has been associated to N-glycosylation of the protein, which does not affect its transport activity suggesting that glutamine import is stimulated in VHL- cells (48). Distinctively, glutamine levels are slightly disturbed by STF-62247 in VHL+ cells, which do not depend on glutamine to survive, and the studied SLC are less affected. On the other hand, protein expression of SLC7A5 was only slightly affected in both STF-treated cells. Instead, a slight but uncharacterized lower band appears only in cells exposed to STF-62247 regardless of VHL genetic background. Because of the role of the SLC7A5/SLC3A2 exchanger to export glutamine and import leucine to activate mTOR, this amino acid was further quantified using LC-MS. Our results showed 50% diminution of leucine levels in VHL- cells treated with the small molecule while it remains stable in VHL+ cells which support the mRNA levels data (**Figure 2D**). Moreover, leucine quantities are similar between both VHL- and VHL+ cells. Finally, the sensitivity of ccRCC to SLC1A5 and SLC7A5 inhibition was tested using pharmacological molecules (**Figure 2E**). GPNA, a widely used SLC1A5 inhibitor, was tested alone or in combination with STF-62247. Results showed that GPNA alone, at 1 mM, significantly decreased cell viability by about 25% in VHL- cells. When GPNA was combined with STF-62247, viability further decreased in VHL- cells supporting the importance of glutamine for these cells. Inhibition of SLC7A5 by BCH did not affect cell viability in both cell lines alone or in combination with STF-62247. Additionally, the results indicated a slight increase of viability in VHL+ cells in response to STF-62247. This effect could be attributed to the use of XTT assay that measures the activity of the mitochondrial dehydrogenase enzymes present in metabolically active cells. In comparison, this effect was not observed using clonogenic assay (**Figure 1C**) or cell counts (**Figures 3C**, **4**) and suggest that NADH, which is produced in the mitochondria to support the complex I of the electron transporter chain is higher in VHL+ treated cells and could contribute to viability of these cells. Overall, the results presented here showed an increase in protein expression of glutamine import transporter SLC1A5 that could reflect an activation by the VHL- cells to compensate for the drop of 70% of glutamine, which reduced its mRNA synthesis and, therefore, indicates that the decrease of glutamine in response to STF-62247 is not likely to be related to SLC1A5 or SLC7A5/SLC3A2.

### Glutamine Uptake Does Not Rescue Cell Viability

We previously demonstrated that ROS production and ATP levels were not affected in response to STF-62247 although a slight increase in ATP was observed in VHL- cells after 48 hr (41, 49). Furthermore, we showed that STF-62247 has no significant effect on mitophagy (41). Nevertheless, we evaluated the rate of mitochondrial oxygen consumption to study another aspect of mitochondrial oxidative pathway (**Figure 3A**). As expected, VHL- RCC4 cells with constitutively active HIF-1α have a lower respiratory control ratio (RCR) compared to VHL+ cells. However, no significant difference on RCR was observed in response to STF-62247 in both cell lines although a slight increase can be observed in VHL-. Thus, to understand the consequence of glutamine diminution on cell survival, an excess of this amino acid was added to the cells exposed to STF-62247. Proliferation and viability curves indicated that VHL- cells and VHL+ control cells were not affected by 10 mM glutamine (**Figures 3B, C**). As anticipated, STF-62247 decrease proliferation in VHL- RCC4 cells, but no change was observed when glutamine concentration was increased. Instead, the results showed that the percentage of viable VHL- cells exposed to STF-62247 trend to further decrease in the presence of 10 mM glutamine while the viability of functional VHL cells was not affected by STF-62247 or by the excess of glutamine. To confirm the internalization of the excess glutamine into the cells, quantification of glutamine and glutamate was performed by LC-MS (**Figure 3D**). Results indicated 3.5 times more intracellular glutamine in VHL- cells when 10 mM glutamine was present in the media. This augmentation was higher in VHL+ cells. However, intracellular levels of glutamate were slightly influenced by the increase of glutamine. When cells were treated with STF-62247, intracellular glutamine and glutamate levels decrease by 42% and 67% in VHL- cells, respectively. The level of these two amino acids was unchanged in cells with the functional VHL gene. Altogether, these results indicate that mitochondrial functions are relatively unaffected by STF-62247. In addition, supplementation of glutamine was not sufficient to rescue cell viability suggesting that glutamine is consumed to support other metabolic functions in response to STF-62247.

### Metabolic Flux of Glutamine in Response to STF-62247

**Figure 5:**
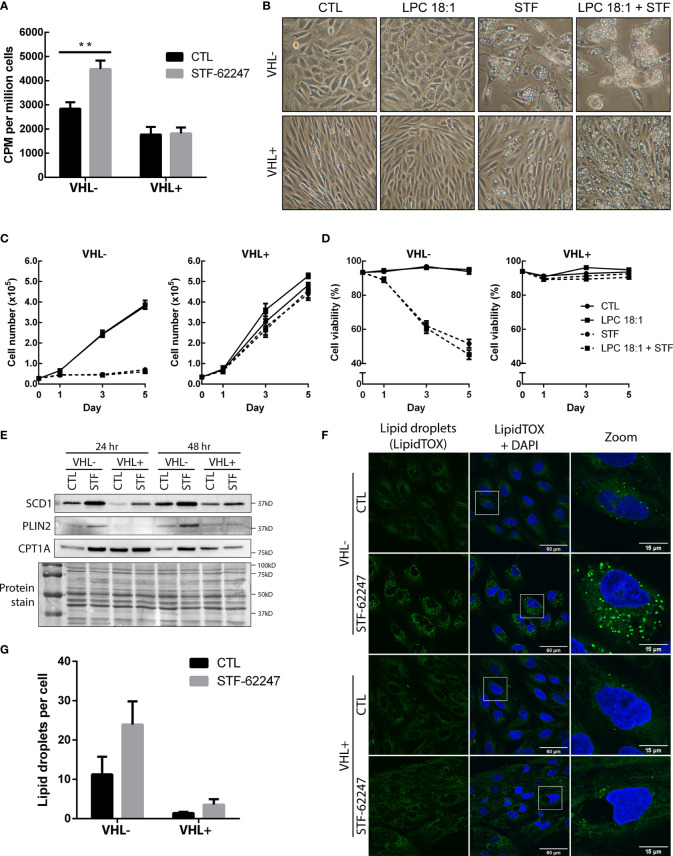
Increase usage of glutamine to produce fatty acids result in the accumulation of lipid droplets in VHL deficient cells. **(A)** Incorporation of radiolabeled carbon (C14) from glutamine into fatty acids in RCC4 VHL- and VHL+ cells after 24 hr of STF-62247 treatment. Culture media was changed for the last hour of treatment with media containing 0,2 μCi/mL (L-[14C(U)]-Glutamine. Radioactivity (counts per minute (CPM)) was measured in lipid extracts from RCC4 VHL- and VHL+ and normalized with the number of cells (N=4). **(B)** Inverted-light microscopy images of vacuolization in RCC4 VHL- and VHL+ cells treated with 1.25 µM STF-62247 and/or 25 µM LPC 18:1 after 3 days. **(C)** Proliferation **(D)** and viability of RCC4 VHL- and VHL+ cells treated with 1.25 µM STF-62247 and/or 25 µM LPC 18:1. Cells were counted on days 0, 1, 3 and 5 with trypan blue to evaluate cells viability (N=3). Statistical marks shown in cell proliferation and viability curves are either between CTL and LPC 18:1, or between STF and STF + LPC 18:1 condition. **(E)** Immunoblot analysis of proteins implicated in fatty acids and lipid droplets metabolism. Cells were treated for 24 hr and 48 hr with 1.25 µM STF-62247. **(F)** Immunofluorescence of fixed RCC4 VHL- and VHL+ cells stained with LipidTOX (green) and DAPI (blue) after being treated for 48 hr with 1.25 µM STF-62247 (N=3). **(G)** Lipid droplets (LipidTOX) puncta **(F)** were analyzed and counted with Fiji (ImageJ). Results are presented as means and SEM of at least three independent experiments. Statistical analyses (Two-Way ANOVA followed by Tukey’s test) were performed to assess significant differences in the results **(A, C, D, G)**. (**p < 0.01).

To better understand the fate of glutamine in STF-treated cells, we used U-^13^C_5_-glutamine to further study intracellular metabolic flux in VHL- cells exposed to the small molecule (**Figure 5A**). Normally, cells using the oxidative TCA cycle are enriched in succinate, fumarate, malate and citrate M+4. However, when cells go into the reductive carboxylation pathway, citrate M+5, malate M+3 and fumarate M+3 are generated. Cells were treated with STF-62247 for 24 hr and then, the media was changed for labeled media. Metabolites were extracted after 0, 20, 60 and 120 min. Labeled glutamine was already well incorporated after 20 min and had reached about 90% of all intracellular glutamine after 120 min (**Figure 5B**). Glutamine levels were also lower in STF-treated cells. Interestingly, levels of succinate M+4, malate M+4 and citrate M+4 were significantly higher in response to STF-62247 (**Figure 5C**). Oppositely, levels of citrate M+5, malate M+3 and fumarate M+3 decreased when cells were treated with STF-62247. Adding to that, the very low levels of α-ketoglutarate M+3, coming from oxidative TCA, show that glutamine is metabolized to form citrate, but that citrate does not continue in the Krebs cycle to generate more α-ketoglutarate. Therefore, independently of the glutamine direction into the Krebs cycle, cells are using glutamine to form citrate. Outside of the Krebs cycle, proline M+5, aspartate M+4, and asparagine, both M+4 and M+3 decreased in response to STF-62247 (**Figure 5C**). Moreover, exogenous asparagine becomes an essential amino acid for cancer cells when glutamine levels decline. We observed a decrease in ASNS protein expression in cells exposed to STF-62247 for 24 hr and 48 hr (**Figure 5D**). Then, proliferation and viability assays were performed using media supplemented with asparagine, but no significant changes were observed in control cells or cells exposed to STF-62247 (**Figure 5E**). These results suggest that while control cells favor reductive carboxylation to produce fatty acid, VHL- cells treated with STF-62247 are using the oxidative TCA cycle to produce citrate.

### Glutamine-Derived Fatty Acid Synthesis and Lipogenesis Are Increased With STF-62247

To determine whether the drop of glutamine can impact FA production, cells were radiolabeled with ^14^C-glutamine and FA synthesis was measured in both cells with functional and non-functional VHL treated with STF-62247. To our surprise, results indicated that FA generated by glutamine was significantly increased in cells exposed to STF-62247 in VHL- cells while no change was observed in VHL+ cells with the functional gene (**Figure 5A**). Then, cells were exposed to an exogenous source of lysophospholipid in the form of lysophosphatidylcholine 18:1 (LPC 18:1), alone or in combination with STF-62247. Increased uptake of LPC is observed in hypoxic cells to sustain proliferation and survival (50). When we observed cell phenotype after 72 hr of treatment, cells were unaffected by treatment with LPC alone. However, when LPC was combined with STF-62247, the size and number of intracytoplasmic vacuoles increased both in VHL- and VHL+ cells (**Figure 5B**). Thus, we evaluated whether the addition of this lipid would affect cell proliferation and viability when treated in combination with STF-62247. However, proliferation and cell viability were not affected by LPC alone or combined with STF-62247 regardless of VHL genetic status (**Figures 5C, D**).

Thus, to study *de novo* lipogenesis and understand the increase of glutamine-derived FA, we evaluated the expression of SCD1, which assures the conversion of saturated FAs into monounsaturated FAs coming from palmitic acid or stearic acid generated from citrate species. Western blot analyses indicated that SCD1 protein is expressed at higher level in RCC4 VHL- cells and increased in response to STF-62247 independently of VHL status (**Figure 5E**). Moreover, FAs and generated products can be stored in lipid droplets (LD) or used for β-oxidation through CPT1A. To further investigate this pathway, PLIN2 expression, a protein associated with LDs, and CPT1A were evaluated. CPT1A is known to be repressed by HIF-α and is highly expressed in VHL+ cells, particularly at 24 hr when confluency is lower (**Figure 5E**). Furthermore, we observed that CPT1A increased, more prominently, in VHL- cells treated with STF-62247. On the other hand, PLIN2 expression is also higher in VHL- cells exposed to STF-62247 while staining of LDs with LipidTOX confirmed these findings (**Figures 5E, F**). Quantification of LDs demonstrated at least two-fold increase in LDs in VHL- cells treated with the small molecule (**Figure 5G**). As expected, PLIN2 and LDs are almost absent from VHL+ cells. Altogether, findings from these studies suggested that intracellular glutamine levels decreased in VHL-mutated cells to sustain lipid demands or storage under this metabolic stress.

## Discussion

Metabolomics studies informed about cancer cell activities by analyzing intracellular metabolites. Major platforms such as 1H NMR, LC-MS/MS and GC-MS have been used to dress a metabolite profiling of RCC mostly in plasma and urine from patient samples (51–53). Using *ex vivo* 1H NMR, a study revealed higher levels of lactate, glutamate, pyruvate, and creatine in RCC tumors compared to normal adjacent tissues and a decrease in acetate, malate, valine, and aspartate (54). In addition, labeling of ccRCC identified reprogrammed metabolic pathways like glycolysis, fatty acid oxidation, and metabolism of amino acids such as tryptophan, arginine and glutamine (24, 51, 55, 56). Besides, metabolomics in cultured RCC cells allows the opportunity to identify changes in metabolites in response to drug treatment or in drug-resistant cells (57). In the present study, we evaluated the effect of a small molecule targeting the loss of VHL on cell metabolism by performing metabolomic profiling using 1H NMR. We observed significant changes in metabolites affected in ccRCC such as valine, creatine, pyruvate, glutathione and glutamate. The drop of glutamate and glutamine observed in VHL- ccRCC cells in response to STF-62247 was particularly interesting since these tumors rely on glutamine for growth and proliferation. In fact, glutaminase inhibitors showed anti-proliferative activity *in vitro* and *in vivo* in mice in a wide range of cancer models including RCC (33, 58, 59). However, clinical trials using GLS inhibitor Telaglenastat (CB-839) combined with the mTOR inhibitor everolimus showed a modest improvement of progression-free survival (PFS) from 1.9 months to 3.8 months while it fails to increase PFS in advanced ccRCC in combination with cabozantinib (33). Our study demonstrated that STF-62247 significantly decreased intracellular levels of glutamine and glutamate without affecting GLS activity, ATP levels, mitochondrial oxygen consumption or mitochondrial membrane potential (data not shown) in VHL- cells. STF-62247 did not significantly change ROS levels and no mitophagy or apoptosis was observed (39, 41, 49). Nevertheless, we were surprised that addition of extracellular glutamine did not rescue, even partly, the viability of VHL- cells in response to STF-62247 due to the importance of this amino acid in RCC metabolic reprogramming. Our results indicated that mitochondria are barely influenced by STF-62247.

Glutamine is taken up in cells mostly through SLC transporters such as SLC1A5. Studies reported high expression of SLC1A5 under hypoxic conditions, which is mediated through HIF-2α, as well as in tumors sample including ccRCC (29, 30, 47). In agreement with these reports, VHL- cells show higher mRNA levels and protein expression of SLC1A5. Glycosylation sites at N163 and N212 are responsible for the localization of SLC1A5 at the plasma membrane and post-translational modifications regulate its stability, trafficking and transport activity (60). Also, a recent paper identified a SLC1A5 variant with a mitochondrial targeting sequence that contribute to ATP production and GSH synthesis (47). Our work did not investigate the effect of STF-62247 on this specific isoform since our findings suggest that mitochondria and related cellular mechanisms are not disturbed by the glutamine drop Instead, we show that STF-62247 increases SLC1A5 protein expression at a very high level compared to VHL+ cells suggesting that this augmentation is to fulfill the glutamine demand. Furthermore, our results by LC-MS/MS with glutamine supplementation suggest that import of glutamine is effective although it was not sufficient to rescue cell viability. We also investigated the expression of SLC7A5 and the chaperone SLC3A2 which work in tandem with SLC1A5 to export glutamine and drive leucine uptake necessary for proliferation and mTORC1 regulation although this mechanism seems to be cell type-dependent (61). SLC7A5 has also been shown to be regulated through HIF-2α (46, 62). Our results showed decrease of the three SLC at the mRNA level in response to STF in VHL- cells while their levels are either unchanged or stimulated in VHL+. This could be explained by the fact that glutamine levels are not affected by the small molecule in VHL+ cells and the SLC are less solicited. On protein expression, we observed an increase of SLC3A2 in cells exposed to STF-62247 but SLC7A5 was mostly unaffected except for the presence of a small band in treated cells. It is surprising that components of this heterodimer are differently expressed although the activity of this transporter can be independent of protein expression. Also, it is known that the chaperone SLC3A2 can also heterodimerize with SLC7A11, but it has not been analyzed in our study. An interesting study demonstrated that SLC7A5 can be internalized by endocytosis, ubiquitinated by the Nedd4-2 ubiquitin-ligase and targeted to the lysosome for degradation (63). The importance of this mechanism remains unknown. Another study showed the localization of SLC7A5/SLC3A2 to the lysosomes, which depend on the protein LAPTM4b to promote the uptake of leucine and mTORC1 activation (45). Our results show significant decrease of leucine in STF-treated VHL- cells, which can be related to mTORC1 inhibition previously reported in VHL-deficient cells (39, 49). It could be interesting to evaluate the localization of these transporters since we showed that STF-62247 accumulates in lysosomes and blocks late-stages of autophagy. We cannot exclude that the drop in leucine might be independent of the coupling system SLC1A5 and SLC7A5/SLC3A2.

Metabolomic flux analysis in presence of labeled glutamine indicated that VHL- cells use the oxidative TCA cycle to produce citrate and maintain energy in response to STF-62247. This metabolic reprogramming could be reflected in the slight increase of mitochondrial oxygen consumption observed in VHL- cells. Since we observed an increase in glucose and pyruvate by NMR in response to STF, it would have been interesting to evaluate the fate of both glutamine and glucose in labeled cells, a limitation of our study. Our metabolomic flux analysis also confirmed the diminution of asparagine and aspartate observed in NMR profiling. Cells can utilize glutamine not only as a source of carbon through the Krebs cycle, but also as a source of nitrogen to produce asparagine *via* the asparagine synthetase (ASNS). This asparagine is generally not used by the cells to fuel the TCA cycle but becomes essential to maintain protein synthesis in tumor cells in deprivation of glutamine (64). Interestingly, in glutamine-deprived cells, asparagine was shown to increase the protein levels of GS, which is important for nucleotide biosynthesis (65). Another study showed that asparagine supplementation alone was sufficient to prevent apoptotic cell death induced by glutamine deprivation (66). Moreover, it has been reported that inhibition of glutaminolysis and depletion of asparagine when autophagy is inhibited causes death in colon cancer cells (67). Our observations indicated that exogenous addition of asparagine or glutamine was not sufficient to prevent VHL-deficient cells from death, although we do see an increase in GS activity. Glutamine and asparagine levels are important to maintain mTOR activities, and deprivation of these amino acids can inhibit mTOR, supporting our previous reports (41, 49, 68).

Alterations in lipid metabolism are often reported in cancer cells such as ccRCC that are characterized by accumulation of lipid droplets. Extracellular uptake of FA is mainly observed in normal cells, but cancer cells prefer to synthesize their own. FA metabolism comprises both catabolism and anabolism of FA. The first one contributes to generating energy through fatty acid oxidation (β-oxidation) and TCA cycle, and the second serves to produce FA from acetyl-CoA and other enzymes such as ATP citrate lyase (ACLY), acetyl-coA carboxylase (ACC) and fatty acid synthase (FASN). Then, FA generated *de novo* are converted into monounsaturated fatty acid by SCD1 and later on in triglycerides and other lipids used for membrane synthesis. Excess lipid can be stored into lipid droplets. Our results demonstrated that VHL- cells utilize glutamine to produce FA as measured using (14)C-glutamine. Thus, we decided to move forward into *de novo* lipogenesis pathway and showed that SCD1 expression is higher in VHL- compared to VHL+ cells. Indeed, a recent report investigated SCD1 protein expression in renal cancer patients using The Cancer Genome Atlas (TCGA) (69). Interestingly, they showed that higher expression of SCD1 was associated with better overall survival and may antagonize the development of more aggressive tumors, which is quite different from other cancer types although another report on chronic myeloid leukemia has similar observations (70). In fact, we showed that STF-62247 increased SCD1 expression in VHL- cells exposed to STF-62247, and not expectingly, in VHL+ cells. Furthermore, we observed an increase of lipid droplets and PLIN2 protein expression in response to STF-62247, both already highly expressed in ccRCC with a loss of VHL. RCC patients with high expression of PLIN2 exhibited better disease-free survival than patients with low levels of PLIN2 (71). In addition, this group also demonstrated that PLIN2 decreases cell proliferation, migration, and invasion, which supports our results on cell survival where VHL- cells exposed to STF-62247 have higher protein expression of PLIN2. The higher level of lipid droplets observed in VHL- compared to VHL+ cells support published paper by Welford group (35). They showed that CPT1A is repressed by HIF-α, which is highly expressed in VHL- cells and causes lipid droplets accumulation in these cells. Interestingly, while we observed increase of lipid droplets, we also showed an increase of CPT1A, which controls FA entry in the mitochondria for β-oxidation, in response to STF-62247 in VHL- cells. Moreover, CPT1A remains stable in VHL+ cells treated to STF-62247. In the same study, they revealed that glucose is necessary for lipid droplets formation in renal cancer cell lines, and not glutamine. This finding was surprising since cells with a loss of VHL are known to depend on glutamine to generate citrate, acetyl-CoA and FA. It would be interesting to elucidate if the rise of glucose observed by NMR in response to STF-62247 is associated with the accumulation of lipid droplets observed in VHL- cells and if, when cells are treated to STF-62247, glutamine can also be used for the formation of the lipid droplets we observed. Overexpression of CPT1A has been reported in other cancer types and normal cells to promote tumor growth and cell proliferation. The increased expression of CPT1A specifically in VHL- cells treated to STF-62247 is quite intriguing since high expression of CPT1A limits tumor progression in VHL-mutated tumors (35). It remains unclear whether increased expression of CPT1A is associated with cell death induced in STF-62247 but this small molecule alters cellular metabolism, particularly glutamine, shown to be involved in the autophagy-lysosome processes. While the identification of STF-62247 intracellular target is still under investigation and points us toward endolysosomal processes, the fate of glutamine in response to STF-62247 helps us to better understand the mechanism of action of this small molecule. Increased levels of key enzymes related to specific lipids could cause perturbations in membrane integrity that can lead to aberrant stress for the cells. We exploit vulnerabilities in ccRCC such as VHL mutations to improve our knowledge of these tumors and develop new types of targeted therapy for patients suffering from this disease.

## Data Availability Statement

The raw data supporting the conclusions of this article will be made available by the authors, without undue reservation.

## Author Contributions

ST conceived and designed the research. MJ performed the experiments presented in **Figures 2C, E**, **3**–**5**. SN performed the experiments presented in **Figures 1D–F**, **Figures 2A, B, D** and **3D**. G.S accomplished **Figures 1G, H**. MT and MC-C realized 1H-NMR analyses. AJ and DB performed LC-MS/MS analyses. DZ performed GC-MS analyses presented in **Figure 5**. M.J prepared the figures. ST and MJ wrote and edited the manuscript. All authors contributed to the article and approved the submitted version.

## Funding

This work was funded by the Canadian Institutes for Health Research (CIHR grant 326436). ST is supported by a research chair from the Canadian Cancer Society (706199). MJ was supported by New Brunswick Innovation Research Assistantship program (RAI 2018-054). SN is supported by a trainee award from the Beatrice Hunter Cancer Research Institute with funds provided by the Cancer Research Training Program and New Brunswick Health Research Foundation.

## Conflict of Interest

The authors declare that the research was conducted in the absence of any commercial or financial relationships that could be construed as a potential conflict of interest.

## Publisher’s Note

All claims expressed in this article are solely those of the authors and do not necessarily represent those of their affiliated organizations, or those of the publisher, the editors and the reviewers. Any product that may be evaluated in this article, or claim that may be made by its manufacturer, is not guaranteed or endorsed by the publisher.
